# Muscle strength in diabetics compared to non-diabetic elderly subjects: A cross sectional and case-control study

**DOI:** 10.22088/cjim.10.3.265

**Published:** 2019

**Authors:** Mohammad Rahimi, Payam Saadat, Seyed reza Hosseini, Mohammad Ali Bayani, Ali Bijani

**Affiliations:** 1 Student Research Committee, Babol University of Medical Sciences, Babol, Iran; 2 Mobility Impairment Research Center, Health Research Institute, Babol University of Medical Sciences, Babol, Iran; 3 Clinical Research Development Unit of Ayatollah Rouhani Hospital, Department of Internal Medicine, Babol University of Medical Sciences, Babol, Iran; 4 Social Determinants of Health Research Center, Health Research institute, Babol University of Medical Sciences, Babol, Iran

**Keywords:** Elderly, Diabetes, Muscle force

## Abstract

**Background:**

With the growing population of the elderly, the prevalence of disabilities and chronic diseases will also likely increase. Muscle weakness leads to low amounts of physical activity in elderly diabetic patients and makes them susceptible to falls. In this study, we aimed to compare the muscle strength between diabetic and non-diabetic elderly individuals.

**Methods:**

The present study is part of the the Amirkola Health and Ageing Project (AHAP) cohort performed on 1320 elderly individuals. Diabetic and non-diabetic subjects were considered as case and control groups, respectively. A diagnosis of diabetes was assigned to patients who were previously diagnosed and those with repeated fasting blood sugar FBS≥126mg/dl. Digi Hand Dynamometer device and manual muscle testing (MMT) grading systems were used to assess muscle force in the upper and lower extremities, respectively. Data were then analyzed and p<0.05 was considered significant.

**Results:**

29.8% of the total participants (n=393) were diabetics. In the case group, 143 (36.4% of all diabetics) had weak upper extremity muscles. The number was 314 (33.9%) among non-diabetics (P=0.38). We saw decreased lower extremity muscle force in 134 (34.1%) diabetic individuals and 292 (31.5%) non-diabetics (P=0.35). Statistical analysis showed no significant difference in any of the lower or upper extremity muscle forces between diabetics and non-diabetics (p>0.05).

**Conclusion:**

Our findings indicate that diabetes mellitus (DM) affects neither the upper nor the lower extremity muscle force in the elderly.

The global prevalence of diabetes mellitus (DM) among people aged more than 65 years has significantly increased during the past two decades (1). Nowadays, roughly one-third of people in this age group have diabetes (2). Muscle weakness increases the risk of diabetes-associated physical disabilities in elderly diabetics and makes them move less (3). Moreover, diabetes is one of the most important factors contributing to low hand grip strength in these patients (4). It is also associated with a higher risk for falls among the elderly (5). Increase in the population of the elderly gives rise to more disabilities and chronic illnesses among these people (6). One of the most remarkable changes in population in the twentieth century is the increase in the number of the elderly (7). Ageing causes physical and biological changes in both the structure and the function of muscles. Muscle mass/intensity deteriorates physically as a result of ageing which leads to a significant reduction of muscle power and function (8). Although no precise mechanism for muscle weakness has been specified in diabetic individuals, studies show that diabetes causes muscle strength degradation in ankle joints through affecting the blood circulation in lower limbs; Other effects include increase in dryness in the dorsiflexion ankle joints and reduction in proprioceptive receptors of these limbs. 

These physical alterations are particular to diabetic individuals and affect their motor activities to a great extent. Although reduction in muscle strength following sarcopenia is quite common among elderlies, it is more prevalent among diabetic elderlies. By increasing the rate of destruction in muscles, hyperglycemia causes deterioration of the muscle mass which leads to a remarkable reduction in muscle strength. 

Decrease in the dorsiflexion range of motion is another consequence of hyperglycemia which is resulted from the increase in the number of collagen fibers with abnormal morphology along with sticky fibers present in the Achilles tendon. Reduction in proprioceptive receptors of ankle affects consistency in walking and consequently raises the risk of falling down while walking (9, 10).

Considering the growing population of the elderly and how diabetes can affect their quality of life, we conducted this study to assess a probable correlation between DM and muscle force among the elderly living in Amirkola, Iran.

Among the practical goals of this study are identification of elderly patients who are suffering from diabetes and are subject to muscle and skeleton weakness and disorders more than others, therefore, prevent their failure and disability in handling daily routine tasks and assist them in preserving their maximum functional power which includes reduction of care expenses and bringing back their health

## Methods

In this study, 393 elderly diabetics were entered into the study as the patient group and 927 non-diabetic elderly individuals were entered as controls. Subject inclusion criteria: 60 and 60+ year old elderlies who already took part in the comprehensive plan Amirkola Health and Aging Project (AHAP) in Amirkola city (11). They signed a written consent prior to their inclusion in the study.

Subject exclusion criteria: Individuals suffering from Parkinson, holding the history of stroke as well as disabled elderlies whose muscular stamina measurability is too low. Also, patients who were not interested in taking part in the study were excluded.

 Subjects were considered diabetic if they had been previously diagnosed with DM or had FBS≥126 mg/dL confirmed by a repeated test. We used Digi Hand Dynamometer device - that measures the force of hand muscles in kilograms - to evaluate the participants’ hand grip. To assess the muscle force in quadriceps, we used the manual muscle testing (MMT) clinical grading system and recorded the results in kilograms. Data from these measurements were then sorted from lowest to highest: The lower third was considered as low muscle force and the remaining was considered as normal. A standard questionnaire, Physical Activity Scale for the Elderly (PASE), was used to assess the amount of physical activity in the study participants. 

The questionnaire comprised three sections with 12 separate questions in each section. Through interview with elder subjects, the questions were answered. Content for questions included activities such as walking, bed resting, activities which entail sitting, and sporting and entertaining activities. The cutoff point was set at 150 for the purpose of data analysis. Sum scores under 150 was regarded as low physical activity and above 150 as reasonable or high level of physical activity (12).

Scores lower than 150 indicated insufficient activity. Depression index was examined in elderlies. Standard Geriatric Depression Scale (GDS) questionnaire was used to collect the related data. GDS comprised of 15 questions based on which the patients were divided into: Normal (scores 0-4), Mild depression (scores 5-8), Moderate depression (scores 9-11), and Severe depression (scores 12-15) (13). Data were collected and then analyzed using SPSS version17 software and statistical tests including logistic regression, chi-squared and t-test. A p<0.05 was considered significant in all analyses. Prior to the sampling, this research project was approved by the Research Ethics Committee of Babol University of Medical Sciences (Ethical registration code: MUBABOL.REC.1394.238). 

## Results

In this study, 393 elderly diabetics were entered into the study as the patient group and 927 non-diabetic elderly individuals as controls (table 1). Among the 393 diabetic participants, 143 (36.6%) had weak upper extremity muscle force. While the stats were somewhat different in the control group (314 out of 927; 33.9%), the difference was not statistically significant (P=0.38). 

With regard to the lower extremity muscle force, the difference between the two groups was not significant either (134 diabetics [34.1%] versus 292 controls [31.5%]; P=0.35) (table 2).

**Table 1:** Frequency distribution and percentage of the participants based on some of the study parameters

**Variable**	**N (%)**
**Gender**	
Male Female	737(55.8) 583 (44.2)
**Age (years)**	
60-64 65-69 70-74 75-79 80-84 80 and older	503(38) 281 (21.3) 233(17.7) 189 (14.3) 77(5.38) 37(2.8)
**BMI (Kg/m** ^2 ^)	
less than 25 25.9-29 30≤	431(32.7) 567(43) 322 (24.4)
**Physical activity**	
<150 >150	1026(77.7) 294(22.3)
**Diabetes**	
No Yes	927(70.2) 393(29.8)
**Hand muscles force**	
Weak Enough	863(65.4) 457(34.6)
**Quadriceps muscle force**	
weak Enough	894(67.7) 426(32.3)

**Table 2:** Correlation between diabetes and upper and lower extremity muscle strengths levels

	**total**	**Diabetes**		**Variables**
		**yes** N (%)	**No** N (%)	
				**Hand muscles force**
0.38	457 863	143(36.6) 250(63.6)	314(33.9) 613(66.1)	Weak Enough
				**Quadriceps muscle force**
0.35	426 894	134(34.1) 259(65.9)	292(31.5) 635(68.5)	Weak Enough

Comparison of diabetics and controls without taking gender into account (table 3) showed a significant difference in variables such as age (P=0.003), upper and lower extremity muscle strengths levels (P=0.008 and P=0.047, respectively), BMI, fasting blood sugar (FBS), weight, existing comorbidities and depression index (all with p<0.001). On the other hand, the amount of physical activity showed no significant difference between the two groups (P=0.56). 

**Table 3:** Comparison of mean and standard deviation of some variables between the diabetic and non-diabetic elderly subjects residing in Amirkola

**Variables**	**Mean**± **SD**	**N**
**Age**		
Non-diabetic Diabetic	69.18 ± 7 67.94 ± 6	927 393
**Hand muscles force**		
Non-diabetic Diabetic	±10 26.85 ± 9 25.26	927 393
**Quadriceps muscle force**		
Non-diabetic Diabetic	10 23.39 ± 10 22.13 ±	927 393
**Body Mass Index ( Kg/M** ^2^ **)**		
Non-diabetic Diabetic	4 26.7 ± ± 4 28.22	927 393
**Fasting Blood Sugar (mg/dl)**		
Non-diabetic Diabetic	11 98.54 ± ± 57 193.94	927 393
**Comorbidities**		
Non-diabetic Diabetic	±1 2.1 ±1 3.4	927 393
**Weight (kg)**		
Non-diabetic Diabetic	13 66.7± ±12 70.31	927 393
**Physical activity**		
Non-diabetic Diabetic	± 61 110.67 ±60 108.53	927 393
**Depression index**		
Non-diabetic Diabetic	± 3 4.1 ± 3 4.8	927 393

Table 4 demonstrates the descriptive and comparative data for diabetic versus non-diabetic men and women. This table shows that the upper and lower extremity muscle force as well as BMI, comorbidities, weight and FBS were all significantly different among the diabetic and non-diabetic men and women. However, comparison of depression index showed no difference between the two groups. Table 5 presents the same descriptive and comparative data in women. Interestingly, we found that among women, diabetes was not associated with upper and lower extremity muscle force. Further evaluation by using logistic regression for multivariate analysis (table 5) revealed no correlation between diabetes and decreased muscle force (OR=1.221; P=0.127 for upper extremity muscle force and OR=1.21; P=0.147 for lower extremity muscle force). 

**Table 4:** Comparison of the mean and standard deviation of some variables between the male and female diabetic and non-diabetic elderly subjects residing in Amirkola

**Variable**	**Male**		**P-value**	**Female**		**P-value**
	**Non-diabetic** **(n=547)**	**Diabetic** **(n=190)**		**Non-diabetic** **(n=380)**	**Diabetic** **(n=203)**	
	**Mean** ±** SD**	**Mean** ±** SD**		**Mean** ±**SD**	**Mean** ±**SD**	
Age	69.62±7	68.52±8	0.078	68.54±7	67.41±8	0.05
Hand muscles force	32.24±8	31.87±9	0.614	19.10±8	19.08±8	0.96
Quadriceps muscle force	28.76± 10	28.39±4	0.661	15.67± 9	16.27±10	0.26
BMI ( Kg/M^2^)	25.66±3	27.19±10	<0.001	28.21±4	29.18±3	0.017
FBS(mg/dl)	97.69±58	166.25±1	<0.001	99.76±10	161.81±58	<0.001
Comorbidities	1.65±1	2.95±1	<0.001	2.9±1	3.9±1	<0.001
Weight (Kg)	67.98±12	73.22±12	<0.001	64.86±12	67.59±12	0.016
Physical Activities	104.63±65	103.39±67	0.824	119.35±65	113.34±67	0.195
Depression Index	3.2±2	3.6±2	0.115	5.4±2	6±2	0.052

**Table 5:** Correlation between upper and lower extremity muscle strengths levels and diabetes considering the other study variables

**Lower extremity muscle strength ** **( Quadriceps)**				**Upper extremity muscle strength** **( Hand)**				
**CI**		**P-value**	**Odds ratio**	**CI**		**P-value**	**Odds ratio**	**Variables**
Upper	Lower			Upper	Lower			
1.565	0.935	0.147	1.21	1.576	0.945	0.127	1.221	Diabetes
0.912	0.469	0.012	0.654	0.941	0.493	0.020	0.665	Body Mass Index
1.038	1.005	0.012	1.021	1.044	1.011	0.001	1.028	Age
1.446	0.889	0.311	1.134	1.624	1.001	0.049	1.275	Sex
1.132	0.633	0.261	0.847	0.893	0.495	0.007	0.665	Physical Activities
**Total**								
**P-value**		**Adjusted OR (CI95%)**		**P-value**		**Crude OR (CI95%)**		
0.261		1.15(0.90-1.47)		0.524		1.08(0.85-1.37)		**Diabetes**
- <0.001 <0.001		- 0.56(0.43-0.73) 0.58(0.42-0.79)		<0.001 <0.001		- 0.60(0.46-0.77) 0.65(0.49-0.87)		Body Mass Index <25 25-29.9 ≥30
0.647		0.95(0.76-1.19)		0.864		0.02 (0.82-1.27)		Age
0.759		0.76(0.60-0.96)		0.122		0.84(0.68-1.05)		Sex(Male)
0.111		0.80(0.62-1.05)		0.137		0.82(0.63-1.07)		Physical Activities>150

## Discussion

Since blood sugar level rises in the elderly, tissue destruction occurs more rapidly that result in a decreased muscle bulk and force. Weaker muscles will lead to less physical activity, which is particularly important in elderly diabetics as it could affect their quality of life and daily activities as well as making them more susceptible to falls (5, 14, 15). We conducted this study on the elderly residing in Amirkola, northern Iran to investigate the correlation between diabetes and muscle force in the extremities. Our results showed no such correlation even after adjustment for other variables. The only exception was that we observed a weaker lower extremity muscle force in diabetic men compared with non-diabetic men- a result that is consistent with some other studies. 

Palacios et al. found no association between muscle weakness and DM in the elderly in their investigation. Moreover, different age and weight distribution among diabetic and non-diabetic groups in their study may have affected this finding (16). Sakkas et al. aimed to evaluate the effect of DM on muscle size and force in patients undergoing dialysis (17). Although the initial raw analysis suggested weaker muscles in diabetic patients compared with controls, multivariate analysis revealed no such correlation. This difference in results was probably because of a higher proportion of women as well as higher mean age and lower physical activity in their patient group. Decreased muscle bulk and higher percentage of fat are both affected by sex, age and physical activity. All in all, they reported no significant difference in muscle force or function between diabetics and controls. 

Similarly, our raw data analysis indicated that both upper and lower extremity muscle forces were significantly lower in the patient group compared with the controls. Furthermore, analysis after adjustment for gender showed no difference in neither upper nor lower extremity muscle force between the two groups. The difference between the results of the two analyses could be due to the presence of both males and females in our study. Women have lower muscle force than men, which could have caused a false correlation in our raw data. Accordingly, our study indicates that female gender is a risk factor for upper and lower extremity muscle force degeneration. Our primary data analysis also revealed a significant difference in age between the two study groups: Mean age was significantly lower among the diabetics compared with non-diabetics. Interestingly, after adjustment for gender, we found that this difference was not significant either and was because the female participants of our study had a lower mean age than our male participants. 

Furthermore, we found that the body mass index (BMI) was significantly higher among diabetics than controls. This finding was confirmed after adjustment for sex, which means this difference was not affected by gender. On the other hand, our findings also demonstrate that increased BMI is associated with lower muscle force in both upper and lower extremities. Thus, overweight and obese elderly individuals with BMI levels of >25 have less risk for developing decreased upper and lower muscle force than those with BMI levels of < 25. This finding is opposed by the study performed by Palacios et al. as they reported subjects with higher BMI levels to be at higher risk of losing their muscle strength (16).

The controversy is further fueled by evidence on both results in several other studies. This inconsistency of findings between studies could be caused by differences in the devices and the criteria used to evaluate muscle strength since there is no unique and globally accepted method to do so (16). This calls for caution in interpreting the results of this study and other similar studies. 

According to our findings, diabetes does not seem to be associated with decreased muscle force in the elderly. Furthermore, the results should be interpreted cautiously as age, sex, BMI and other conditions and comorbidities could affect them all and cause controversial findings in different studies. 

## References

[1] Nejati V, Ashayeri H (2008). Health related quality of life in the elderly in Kashan. Iran J Psychiatry Clin Psychol.

[2] Bweir S (2014). Relationship between gait deviations and risk of falls in patients with type 2 diabetes. Eur Sci J.

[3] Balducci S, Sacchetti M, Orlando G (2014). Correlates of muscle strength in diabetes: the study on the assessment of determinants of muscle and bone strength abnormalities in diabetes (SAMBA). Nutr Metab Cardiovasc Dis.

[4] Park SW, Goodpaster BH, Strotmeyer ES (2006). Decreased muscle strength and quality in older adults with type 2 diabetes: the health, aging, and body composition study. Diabetes.

[5] Ng TK, Lo SK, Cheing GL (2014). The association between physical characteristics of the ankle joint and the mobility performance in elderly people with type 2 diabetes mellitus. Arch Gerontol Geriatr.

[6] Bayani MA, Karkhah A, Hoseini SR (2016). The relationship between type 2 diabetes mellitus and osteoporosis in elderly people: a cross-sectional study. Int Biol Biomed J.

[7] Sadeghiyan F, Raei M, Hashemi M, Amiri M, Chaman R (2011). Elderly and health problems: a cross sectional study in the shahroud township. Iran J Ageing.

[8] Hairi NN, Cumming RG, Naganathan V (2010). Loss of muscle strength, mass (sarcopenia), and quality (specific force) and its relationship with functional limitation and physical disability: the concord health and ageing in men project. J Am Geriatr Soc.

[9] Vinik AI, Vinik EJ, Colberg SR, Morrison S (2015). Falls risk in older adults with type 2 diabetes. Clin Geriatr Med.

[10] Ng TK, Lo SK, Cheing GL (2014). The association between physical characteristics of the ankle joint and the mobility performance in elderly people with type 2 diabetes mellitus. Arch Gerontol Geriatr.

[11] Ferns GA, Ghayour-Mobarhan M (2018). Metabolic syndrome in Iran: a review. Translational Metab Synd Res.

[12] Ishaghi R, Mahmoudian SA, Asgarian R (2011). Effect of faith-based education on physical activity on the elderly. Iran J Med Educ.

[13] Yesavage JA, Sheikh JI (1986). 9/Geriatric depression scale (GDS) recent evidence and development of a shorter version. Clin Gerontol.

[14] Smith LA, Branch LG, Scherr PA (1990). Short‐term variability of measures of physical function in older people. J Am Geriatr Soc.

[15] Kalyani RR, Saudek CD, Brancati FL, Selvin E (2010). Association of diabetes, comorbidities, and A1C with functional disability in older adults: results from the National Health and Nutrition Examination Survey (NHANES), 1999-2006. Diabetes Care.

[16] Palacios-Chávez M, Dejo-Seminario C, Mayta-Tristán P (2016). Physical performance and muscle strength in older patients with and without diabetes from a public hospital in Lima, Peru. Endocrinol Nutr.

[17] Sakkas GK, Kent-Braun JA, Doyle JW (2006). Effect of diabetes mellitus on muscle size and strength in patients receiving dialysis therapy. Am J Kid Dis.

